# Accuracy and Safety of Robot‐Assisted Drilling Decompression for Osteonecrosis of the Femoral Head

**DOI:** 10.1111/os.12678

**Published:** 2020-05-11

**Authors:** Jin Luo, Ya‐jing Yan, Xiao‐dong Wang, Xu‐dong Long, Hai Lan, Kai‐nan Li

**Affiliations:** ^1^ Department of Orthopaedics Affiliated Hospital of Chengdu University Chengdu China

**Keywords:** Borehole decompression, Femoral head necrosis, Orthopaedic surgery robot positioning system

## Abstract

**Objective:**

To investigate the safety and superiority of robot‐assisted femoral head drilling decompression in the treatment of femoral head necrosis.

**Methods:**

A total of 63 patients who underwent borehole decompression of the femoral head in our hospital from January 2016 to March 2019 were recruited. Patients were divided into two groups for comparison according to surgical methods. In the robot‐assisted surgery group, there were 30 cases with 41 femoral heads. The conventional group had 33 cases and 46 femoral heads. All patients signed the consent form before the operation. The follow‐up time was 6 months. The incision lengths, operation times, intraoperative blood loss, intraoperative fluoroscopies, guide needle punctures, postoperative Harris scores, and postoperative complications of the two groups were compared.

**Results:**

The incision length of the robot surgery group was 5.16 ± 0.41 cm, while that of the traditional surgery group was 7.42 ± 0.50 cm. The operation time of the robot surgery group was 46.99 ± 4.94 min, while that of the traditional surgery group was 55.01 ± 6.19 min. The fluoroscopy frequency of the robot surgery group was 10.50 ± 1.78 times, while that of the traditional surgery group was 17.91 ± 2.20 times. The intraoperative blood loss in the robotic surgery group was 20.62 ± 2.52 mL, while that in the conventional surgery group was 52.72 ± 3.39 mL. In the robot operation group, each femoral head guide needle was punctured three times, and the puncture was successful one time. The number of guided needle punctures in the traditional group was 8.02 ± 1.73. The difference between the two groups was statistically significant (*P* < 0.05). The Harris score was 69.53 ± 7.51 in the robot surgery group and 68.38 ± 7.26 in the traditional surgery group one month after surgery, 78.52 ± 6.49 in the robot surgery group and 76.41 ± 7.95 in the traditional surgery group three months after surgery, and 83.32 ± 8.62 in the robot surgery group and 81.74 ± 6.20 in the traditional surgery group six months after surgery. There was no significant difference between the two groups (*P* > 0.05). In the traditional group, there was one case of incision infection and one case of femoral head collapse during follow‐up. In the robot group, there were no complications, such as incision infection and deep vein thrombosis. No collapse of the femoral head was found in the robot group during follow‐up.

**Conclusion:**

The positioning system of the orthopaedic robot is an ideal method for the treatment of femoral head necrosis. This method has the advantages of simple operation, accurate drilling, a short operation time, less surgical trauma, less radioactivity, and good recovery of hip joint function.

## Introduction

Osteonecrosis of the femoral head (ONFH) is also called avascular necrosis and aseptic necrosis. It is a common disease in orthopaedics^1^, ^2^ that has a high disability rate and lacks effective treatment in clinical practice^3^. There are many causes of femoral head necrosis, which are mainly divided into two categories: traumatic and non‐traumatic^4^. The main causes of nontraumatic femoral head necrosis in China are corticosteroid application, alcohol abuse, decompression sickness, sickle cell anaemia, and idiopathic type^5^, ^6^. The most common causes are glucocorticoids, alcohol, and trauma. Pathologically, the density of functional microvessels in the subchondral bone of the diseased femoral head decreases, the vascular permeability is abnormal, and the angiogenesis ability becomes poor. Bone formation‐related apoptosis of the diseased femoral head increases, and progenitor cell proliferation and osteogenic differentiation are decreased. In addition, there is hypertrophy of adipocytes along with infiltration of inflammatory cells in the diseased femoral head. The imbalance of the bone formation system can be further manifested as sparsity, fracture, and microfracture of trabecular bone. The dissection of cartilage and subchondral bone can be observed by gross pathology. Moreover, the vascular lesions and imbalanced bone formation in ONFH affect each other^7^, ^8^, ^9^.

ONFH occurs early and leads to collapse of the femoral head and dysfunction of the hip joint in relatively young patients, who then ultimately require total hip replacement surgery, resulting in a decline in personal and social productivity^10^. Although hip arthroplasty is currently one of the most successful operations in the field of bone science, the associated complications (including infection, prosthesis loosening, dislocation, and periprosthetic fractures) are increasingly prominent. The limited life and high cost of joint prostheses restrict their wide application. Patients have to endure great physical pain and financial burden. Therefore, the search for safe, effective, and minimally invasive treatment has always been the focus of orthopaedic research^11^. Core decompression (CD) can reduce the pressure in the femoral head, open the hardened areas that hinders the repair of osteonecrosis, stimulate the formation of blood vessels around the decompression tunnel, enhance the replacement of new bone, and delay the progress of osteonecrosis^12^. The effect of CD on femoral head necrosis is definite. However, in the operation, how to operate in a more minimally invasive manner as well as more correctly and more accurately is still worth exploring.

With the development of science and technology, orthopaedic robots have gradually developed, promoting the development of minimally invasive surgery. Robots were first used in brain surgery in the 1980s. Surgical robots have the characteristics of flexible operation, good stability, strong hand‐eye coordination and accurate movement. Such robots also have the advantages of repeatability and fatigue resistance, which can break through the limitation of doctors' freehand ability and further improve the accuracy and safety of surgery. Surgical robots are increasingly being used in clinical treatments and were first used in orthopaedics in 1992. According to the technical characteristics and application patterns, orthopaedic surgical robots are divided into autonomous, tactile, and passive devices. They are commonly used in spinal surgery, joint replacement surgery, and orthopaedic trauma surgery for hips and pelvis. Compared to conventional hands‐only screw placement, robot‐assisted pedicle screw placement has been shown to significantly reduce the risk of nerve damage during spinal surgery.

At home and abroad, orthopaedic surgical robots have developed rapidly, and there are many types of robots that are widely used in orthopaedic surgery. However, in China, robot‐assisted surgery is still in its infancy. In 2015, Beijing Tianzhihang Medical Technology Co., Ltd. successfully developed the third generation of orthopaedic surgical robots. This robot has a 6‐degree‐of‐freedom series manipulator arm with an arm length of more than 800 mm. The robot has the function of active positioning and human‐computer cooperative movement. It can achieve safe and accurate surgical positioning by combining the rough positioning performed by surgeons and the precise positioning of the robot active positioning function.

It has been reported that compared to traditional surgery, orthopaedic robot‐assisted surgery can reduce surgical time, reduce radiation exposure time, make operations more accurate, and reduce surgical trauma^13^, ^14^. Robots are considered a potential solution to overcome the defects of traditional surgery and have quickly become the focus of current research. Furthermore, they play an important role in assisted surgery. There are still many shortcomings in the application of orthopaedic surgery robots. Unlike a human, an orthopaedic surgical robot does not have a tactile sensory feedback system during the operation, so it cannot well prevent iatrogenic injuries in patients, and its scope of application is relatively effective. In addition, at present, the orthopaedic surgical robot is relatively large and inconvenient to operate. In sum, these are issues that clinicians and robot researchers urgently need to solve. In the future, orthopaedic surgery robots will be developed with an aim towards low cost, miniaturization, and specialization^15^. However, with the development of precision medicine and minimally invasive surgery, orthopaedic robot navigation assistance technology has been widely applied in orthopaedic surgery due to its advantages in accuracy, rapidity, safety, and other aspects.

The purposes of this study are as follows: (i) to compare the clinical efficacy of the orthopaedic robot positioning system in assisting decompression of the femoral head and decompression of the femoral head in treating femoral head necrosis; and (ii) to explore the superiority of the orthopaedic robot positioning system in assisting femoral head decompression to treat femoral head necrosis.

## Materials and Methods

### *Inclusion and Exclusion Criteria*

#### *Inclusion Criteria*

The inclusion criteria were as follow: (i) all patients were diagnosed with femoral head necrosis through medical history, symptoms, physical examination, and magnetic resonance imaging (MRI); (ii) all patients underwent robotic‐assisted femoral head decompression surgery or traditional femoral head decompression surgery; (iii) regarding the staging of femoral head necrosis, all patients were Ficat stage I and Ficat stage II; and (iv) the main evaluation indicators included surgical incision length, surgical time, intraoperative blood loss, intraoperative perspectives, number of punctures, and Harris score. This study was a retrospective case‐control study.

#### *Exclusion Criteria*

The exclusion criteria were as follows: (i) patients diagnosed with Ficat III and Ficat IV stage disease; (ii) patients who had necrosis of the femoral head due to glucocorticoids and the need to continue to use glucocorticoids; (iii) patients who could not tolerate general anaesthesia due to systemic conditions such as heart and lung disease; (iv) patients with systemic infection or local skin infection around the incision; (v) patients with disease combined with tumours; (vi) patients with mental illness; and (vii) patients who rejected surgery.

### *General Information of Participants*

From January 2016 to March 2019, 63 patients in our hospital underwent borehole decompression of the femoral head. Patients were divided into two groups for comparison according to the surgical methods. In the robot‐assisted surgery group, there were 30 patients with 41 femoral heads, including 18 males and 12 females, aged from 29 to 65 years, with an average age of 53 years. In the traditional surgery group, there were 33 cases with 46 femoral heads, including 20 males and 13 females, aged from 29 to 65 years, with an average age of 50 years. All patients signed the consent form before the operation. The general situation of the patients is shown in Table 1. There was no significant difference in gender, age, or Harris score before surgery between the two groups, and they were comparable (*P* > 0.05).

**Table 1:** Basic information of the two groups of patients

Groups	Number of cases		Gender		Age (years, mean±SD)	Harris score before surgery (mean±SD)
	Unilateral	Bilateral	Male	Female		
Robot group	19	11	18	12	53.00±7.09	65.17±6.92
Traditional group	20	13	17	16	50.00±8.84	64.42±5.90
Statistics value	0.050		0.458		1.474	0.464
*P* value	0.824		0.498		0.146	0.644

**Figure 1:**
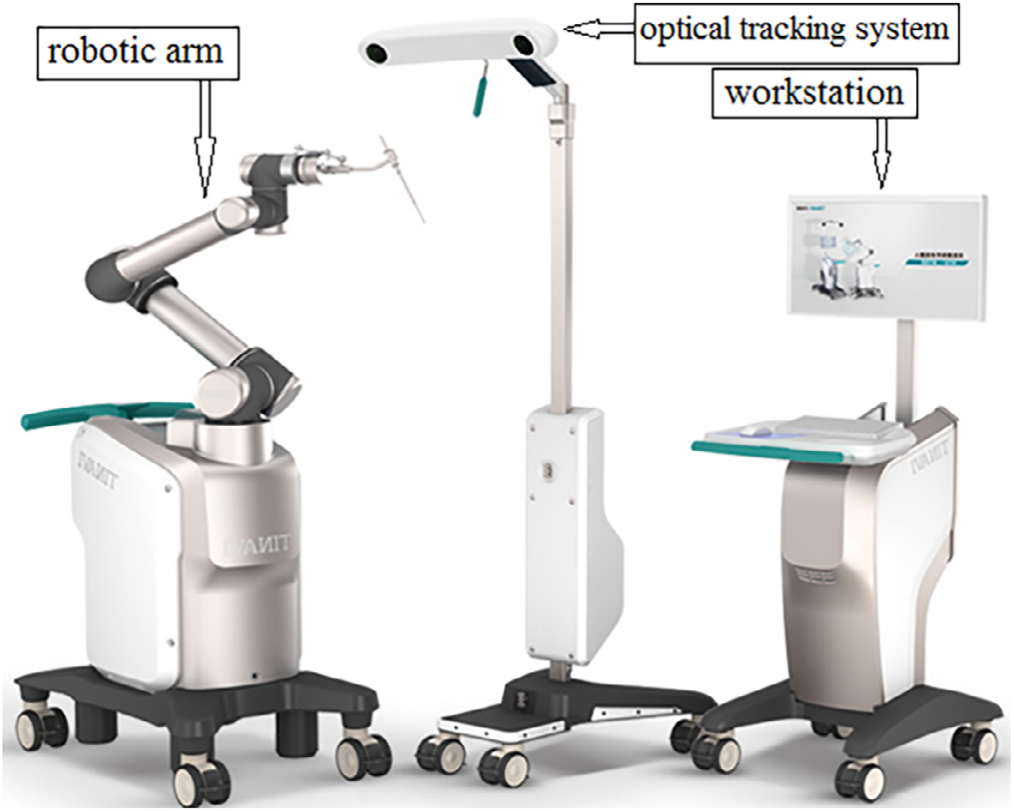
TiRobot, mainly composed of a workstation, an optical tracking system, and a robotic arm (Photo provided by Beijing Tianzhihang Medical Technology).

The orthopaedic robot navigation surgery group performed the operation with the help of the third‐generation orthopaedic surgical robot TiRobot of Beijing Tianzhixing Medical Technology (Fig. 1).

### *Surgical Methods*

#### *Orthopaedic Robot Navigation‐Assisted Drill Decompression of the Femoral Head*

##### Preoperative Preparation of the Robot

Before the operation, it was first checked whether the robot equipment was complete. According to the operation instructions of the TiRobot orthopaedic robot system, the robot system was installed and debugged by referring to the method of drilling and decompressing the femoral head assisted by the robot navigation and positioning system. The workstation, C‐arm X‐ray machine, manipulator, and optical tracking system equipment were arranged in coordination with routine preoperative preparation. The power was turned on, the device was connected, and it was checked whether the device was working properly. Finally, the surgeon logged on to the system, recorded the medical records, and selected the surgical tools.

##### Surgical Methods of the Robot Group

**Figure 2:**
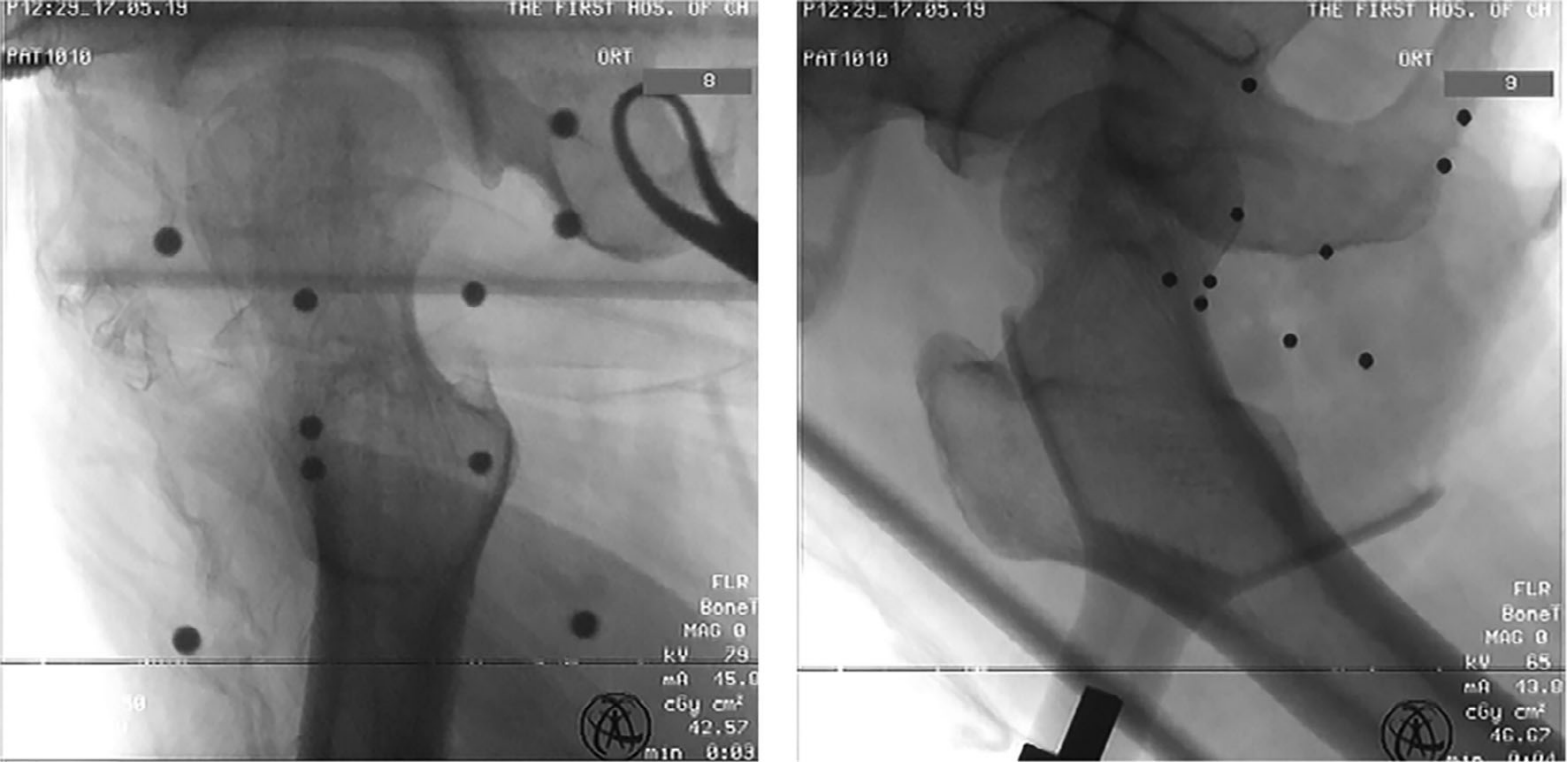
The image of the hip joint in the positive and lateral position. All ten anchor points appear in the upper portion of the image.

The patient was placed in the supine position on a traction bed with both lower limbs abducted. The surgical area was routinely sterilized and covered with a sterile surgical towel to fully expose the surgical area on the affected side and the anterior superior iliac spine, and a tracer was placed on the affected side at the site of the anterior superior iliac spine. A sterile C‐arm pocket was placed on the robot arm, and the positioning ruler was calibrated and adjusted to the proper position. A C‐arm X‐ray machine was used to observe the X‐rays of the hip joint in the upright position and the lateral position. All 10 positioning points on the positioning ruler were included in the X‐ray image (Fig. 2). The images acquired by the C‐arm X‐ray machine perspective were imported into the workstation. The position of each anchor point was determined and numbered. Each positioning point was fine tuned to ensure that it was in the best position, thereby reducing errors. The surgical path was planned, and the appropriate nailing point, angle, and length were designed on the workstation.

**Figure 3:**
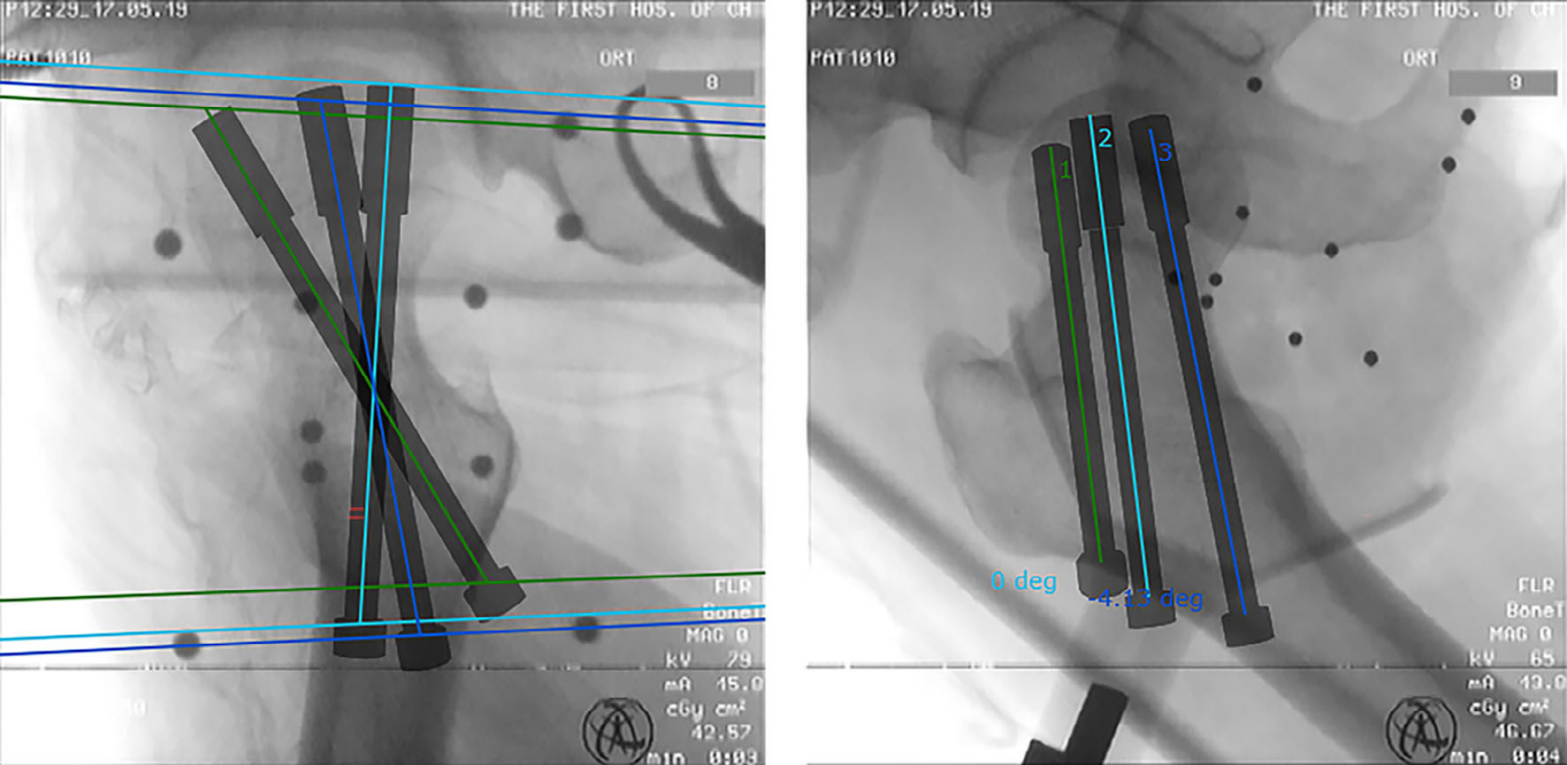
The placement planning path and simulation graph of the guide wire were designed according to the positive and lateral images of the hip joint imported into the workstation.

The positioning ruler was replaced in front of the robot arm with the guide needle cannula. The computer was used to simulate the movement process of the robot arm on the operation platform, and the movement process and direction were confirmed to be correct. Then, the robot arm was run. The robotic arm automatically moved the guide needle cannula to the skin surface according to the planned direction and needle insertion point. The guide needle was inserted into the patient through this point and direction. The surgeon made an incision of approximately 1–2 cm in length at the skin where the needle was inserted (Fig. 3). Soft tissue was bluntly separated, and a Kirschner wire was placed along the guide cannula. According to the position of the femoral head measured by the robot, an appropriate length of the Kirschner wire was drilled. At this time, the Kirschner wire was firmly fixed in the bone. Then, a 3.2 mm diameter hollow drill was inserted in the direction of the Kirschner needle to below the bone cortex. After the length and position were satisfied, the Kirschner needle and the hollow bit were pulled out. Two other Kirschner needles were inserted in this way to decompress the femoral head. It should be noted that the depth of the Kirschner wire could not enter the joint cavity. The wound was rinsed, the bleeding was completely stopped, and the incision was closed.

#### *Traditional Surgical Methods*

The patient was placed in the supine position on a traction bed, with both lower extremities abducted on traction beds. C‐arm X‐ray radiography of the hip joint and the body surface location of the greater trochanter marker bone was performed. The longitudinal incision of the lateral hip joint along the lower margin of the greater trochanter of the femur was approximately 6–9 cm long. The subcutaneous fascia and muscles were bluntly separated up to the periosteum. The C‐arm fluoroscopy was performed again to confirm the drilling direction of the Kirschner needle. A Kirschner wire was inserted into the bone at an appropriate length, and then a 3.2 mm diameter hollow drill was used to drill into the femoral head below the femoral cortex. The Kirschner wire was prevented from penetrating the femoral head into the articular cavity. Then, the Kirschner needle and hollow bit were exited with satisfactory depth of the C‐arm perspective hole. Repeated drilling was performed three times in different needle directions to fully decompress the femoral head. The wound was rinsed, the bottom was evaluated for haemostasis, and the incision was closed.

### *Postoperative Treatment*

After the operation, pain relief, energy supplementation, electrolyte supplementation, acid and base balance maintenance, internal environment stabilization, and dressing changes should be administered. X‐ray films were reviewed after the operation, and MRI was reviewed 6 months after the operation. On the second day after surgery, the robot group began to get out of bed and walk under a semi‐weight‐bearing state. Patients in the traditional group started to move with the help of crutches 3 days after surgery. One month after the operation, the crutch load was removed gradually.

### *Observation Indicators*

#### *Intraoperative Incision Length*

The length of the incision created during surgery is determined according to the actual requirements of the operation. To satisfy the intraoperative operating field of vision, the smaller the cut length is, the better. Small incisions can affect the accuracy and speed of intraoperative operations. Large incisions can increase the amount of intraoperative blood loss and the length of the healed scar, which goes against the concepts of minimal invasiveness, accuracy, and aesthetics in contemporary medicine.

#### *Operation Time*

The operating time is calculated from the time the skin is sterilized and the sterile towels are placed until the skin is stitched. The time of the operation is influenced by many factors, such as the proficiency of the operator, the number of C‐arm fluoroscopies, and the tacit cooperation between the operator and the assistant.

#### *Intraoperative Bleeding Volume*

Intraoperative haemorrhage was collected by drainage bags. The length of the operation and the length of the incision will affect the amount of bleeding.

#### *Number of Intraoperative Fluoroscopies*

We recorded the number of fluoroscopies during the operation. The number of fluoroscopies is affected by the number of punctures and the puncture accuracy. The more fluoroscopy patients and doctors receive, the more radiation they are exposed to.

#### *Number of Guide Needle Punctures*

We recorded the number of needle punctures. The puncture number is affected by the puncture accuracy of the guide needle. The number of guide needle punctures can be used to evaluate the accuracy of the operator in the operation.

#### *Harris Score*

The Harris score can be used to evaluate the recovery of postoperative hip joint function. All patients were evaluated for hip function 1 month, 3 months, and 6 months after surgery. Hip function was evaluated in all patients according to the Harris score and included pain, function, range of motion, and degree of deformity.

#### *Complications*

Common complications included infection of the incision, deep vein thrombosis of the lower extremity, and collapse of the femoral head. The number of complications was also an important auxiliary observation index to evaluate the operation.

### *Statistical Methods*

All data were statistically analysed by the statistical software IBM SPSS 22.0 (International Business Machines Corporation, Armonk, New York, USA). Quantitative data included surgical incision, operative time, intraoperative fluoroscopy, intraoperative puncture, intraoperative blood loss, and Harris score. The inter‐group measurement data were tested by the K–S normal distribution test and variance homogeneity test. If the data conformed to a normal distribution and the variance was neat, the measurement data between the groups were analysed by the *t* test. All data were expressed as the mean ± standard deviation. Count data included gender and the number of unilateral and bilateral cases between groups. Comparisons between counts were performed using χ^2^‐test analysis. *P* < 0.05 was considered statistically significant.

## Results

The surgical incision length, operation time, number of intraoperative fluoroscopies, intraoperative blood loss, and Harris score of the two groups were consistent with a normal distribution. Intraoperative results are shown in Table 2, and Harris scores are shown in Table 3. The number of puncture times of the guide needle does not conform to the normal distribution, and the average value is directly compared.

**Table 2:** Intraoperative data results (mean±SD)

Groups	Number of femoral head	Surgical incision length (cm)	Operating time (min)	Intraoperative fluoroscopy times	Intraoperative bleeding volume (mL)
Robotic surgery group	30 (41)	5.16 ± 0.41	46.99 ± 4.94	10.50 ± 1.78	20.62 ± 2.52
Traditional surgery group	33 (46)	7.42 ± 0.50	55.01 ± 6.19	17.91 ± 2.20	52.72 ± 3.39
*t* value		−22.95	−6.63	−17.16	−49.56
*P* value		<0.001	<0.001	<0.001	<0.001

**Table 3:** Harris score at postoperative follow‐up (mean±SD)

Groups			
	1 month	3 months	6 months
Robotic surgery group	69.53 ± 7.51	78.52 ± 6.49	83.32 ± 8.62
Traditional surgery group	68.38 ± 7.26	76.41 ± 7.95	81.74 ± 6.20
*t* value	0.619	1.144	0.838
*P* value	0.538	0.257	0.405

#### *Surgical Incision Length*

The incision length of the robot surgery group was 5.16 ± 0.41 cm, while that of the traditional surgery group was 7.42 ± 0.50 cm. Compared with the traditional surgery group, the incision length of the robot surgery group was shortened by 30.46%. The difference between the two groups was statistically significant (*P* < 0.05).

#### *Operation Time*

The operation time of the robot surgery group was 46.99 ± 4.94 min, while that of the traditional surgery group was 55.01 ± 6.19 min. Compared with the traditional group, the robotic surgery group reduced the operation time by 14.58%. The difference between the two groups was statistically significant (*P* < 0.05).

#### *Number of Intraoperative Fluoroscopies*

The fluoroscopy frequency of the robot surgery group was 10.50 ± 1.78 times, while that of the traditional surgery group was 17.91 ± 2.20 times. Compared with the traditional surgery group, the number of intraoperative fluoroscopies in the robot surgery group reduced by 41.37%. The difference between the two groups was statistically significant (*P* < 0.05).

#### *Intraoperative Bleeding Volume*

The intraoperative blood loss in the robotic surgery group was 20.62 ± 2.52 mL, while that in the conventional surgery group was 52.72 ± 3.39 mL. Compared with the traditional surgery group, the intraoperative bleeding volume in the robot surgery group was reduced by 60.89%. The difference between the two groups was statistically significant (*P* < 0.05).

#### *Number of Guide Needle Punctures*

In the robot operation group, each femoral head guide needle was punctured three times, and the puncture was successful one time. However, in the traditional operation group, the number of punctures for each femoral head guide needle were ≥3; the highest was 12, and the average was 8.02 ± 1.73. There were significant differences between the two groups.

#### *Harris Score*

The Harris score was 69.53 ± 7.51 in the robot surgery group and 68.38 ± 7.26 in the traditional surgery group 1 month after surgery. There was no significant difference between the two groups (*P* > 0.05). The Harris score was 78.52 ± 6.49 in the robot surgery group and 76.41 ± 7.95 in the traditional surgery group 3 months after surgery. There was no significant difference between the two groups (*P* > 0.05). The Harris score was 83.32 ± 8.62 in the robot surgery group and 81.74 ± 6.20 in the traditional surgery group 6 months after surgery. There was no significant difference between the two groups (*P* > 0.05).

#### *Complications*

In the traditional group, there was one patient with infection of the surgical incision, which was cured after antibiotic treatment and dressing change. One patient experienced femoral head collapse during the follow‐up period, but this patient asked for conservative treatment without any operation. In the robot group, there were no surgical complications, such as incision infection and deep vein thrombosis. No collapse of the femoral head was found during the follow‐up period.

## Discussion

ONFH is a common orthopaedic disease, also known as ischemic femoral head necrosis, which is more common in young adults and can be divided into two categories: traumatic and nontraumatic. At present, in the early stage of ONFH, clinicians mainly confirm the diagnosis through computed tomography (CT) and MRI^16^, ^17^. The accuracy of MRI is high, and the specificity and sensitivity of MRI for early detection of osteonecrosis is up to 99%, and the condition is thus not easy to miss and be misdiagnosed^18^.

The treatment of ONFH has always been a concern of clinicians. Femoral head drill decompression was pioneered by Arlet and Float in 1964. Borehole decompression of the femoral head is an effective treatment that has been widely used by clinicians at home and abroad^19^. At present, the procedure has been improved, and some scholars believe that multiple orifice decompression is more conducive to the treatment of the femoral head. In this study, only one patient in the traditional group had femoral head collapse, while the number of cases in the robot group was zero. This result is better than that of Zhao *et al*.^20^. One reason may be that the follow‐up time of this study was short. The other reason is that the surgical method used in this study was different. Zhao *et al*.’s operation involved drilling a large hole in the centre of the femoral head, while in this study, multiple holes were drilled. The trabecular bone between each hole can support the femoral head and prevent it from collapsing. However, traditional femoral head decompression surgery involves a long incision, substantial bleeding, relatively serious damage to the muscles and ligaments around the intertrochanteric femur, and a long postoperative recovery time.

With the development of the concept of accelerated rehabilitation and minimal invasiveness, doctors and patients have increasingly higher requirements for the safety and effectiveness of treatment technology. Orthopaedic surgeons are increasing their research on reducing patient trauma. Our hospital is already familiar with the operation procedures of orthopaedic robots and has achieved good results^21^. TiRobot, which was independently developed in China, is the latest advanced orthopaedic robot system^22^. The robot system adopts modularization, miniaturization, and a general design to realize the breakthrough of operation platform technology. In traumatic orthopaedic internal fixation surgery, the orthopaedic robot can accurately assist doctors in locating implants, with a positioning accuracy of 0.6–0.8 mm. Minimally invasive surgery and high‐risk regional surgery have obvious advantages under the guidance of orthopaedic surgery robots, which can reduce the complexity of surgery and effectively reduce the risk of surgery^23^.

In this study, TiRobot‐assisted drilling and decompression of the femoral head under general anaesthesia showed satisfactory results in the treatment of femoral head necrosis. TiRobot can precisely guide the surgeon to the predetermined position of the operation and determine the exact direction of puncture, making the operation simpler, more accurate, and less traumatic^24^, ^25^. It has four main features. First, the robot can provide accurate spatial positioning and a stable insertion path. Through the movement of the robotic arm, the screws are placed in the corresponding anatomical site accurately, safely and stably. The second feature is reduced operation time. Real‐time optical tracking technology eliminates the need for repeated X‐rays during surgery, increasing the flexibility and smoothness of the operation, shortening the operation time, and improving the operation efficiency. The third feature is reduced radiation damage. Compared with traditional surgical operations, robotic navigation significantly reduced the number of intraoperative X‐ray examinations, thereby significantly reducing the cumulative intraoperative radiation dose. The final feature is procedural surgery. During the operation, the surgical plan and route positioning are completed through the robot system to guide the doctors in completing the operation effectively and safely.

At the beginning of the application of orthopaedic robots, more time was spent on robot preparation and preoperative planning, probably because the operation process was not very familiar^26^. In our hospital, the reduction in femoral head pressure by drilling assisted by orthopaedic robots is compared with that by drilling in traditional ways. In our hospital, a comparative analysis was performed between drilling decompression of the femoral head assisted by an orthopaedic robot and traditional drilling decompression of the femoral head. Robot‐assisted surgical incisions, intraoperative blood loss, number of fluoroscopies, number of guide needle punctures, and operation time were better than those of the traditional group. Traditional drilling and decompression surgery methods require large incisions, usually requiring 4–10 cm, extensive damage to the surrounding tissues, and a long recovery time. When inserting a hollow drill, it is necessary to repeat the C‐arm fluoroscopy to confirm the accuracy of the drill bit direction, thereby increasing the radiation exposure of the patient and the operator, as well as the surgical time. Because it is difficult to determine the direction of the borehole in the traditional surgical method, it is easy to cause repeated punctures of the Kirschner needle.

According to statistics, the dislocation rate of guide wire used under fluoroscopy guidance has been reported to be between two and 15^27^, ^28^. Robot‐assisted orthopaedic surgery is thought to have the potential to improve implant placement accuracy and reduce radiation and surgical time^29^. This result is consistent with the findings of this study. Robot‐assisted surgery requires only a small incision, not a long incision, to expose the greater trochanter. There is no gluteus medius overdissection, which reduces the incidence of postoperative hip abductor strength loss. Therefore, robot‐assisted surgery can effectively reduce the amount of blood loss in patients and the degree of surgical trauma so that the operation is safer, more effective, and conducive to fracture healing and early postoperative rehabilitation exercise. The difference between the two groups of patients was statistically significant. Robotic‐assisted decompression was performed on the femoral head with a small incision. Generally, only 1–2 cm is needed for one hole, with less bleeding and quick postoperative recovery. This is one of the advantages of this procedure.

In addition, when drilling three times, the skin incisions required by the holes in different directions are spaced apart, which is more conducive to the recovery of the incision. Especially for young patients, in order to delay or avoid the occurrence of joint replacement as much as possible, and to maintain the beauty of the skin, this operation is a better choice. Indeed, it can delay hip replacement and achieve minimally invasive requirements. However, due to the short follow‐up time of this group of patients, it is necessary to conduct longer follow‐ups and summarize more comprehensive follow‐up data to determine the exact effect of this operation.
